# Multimodal physiotherapeutic management of postoperative complications in orthognathic surgery: A literature review and institutional protocol proposal

**DOI:** 10.4317/medoral.27687

**Published:** 2025-10-17

**Authors:** Alejandro Fernández-de-la-Reguera, Alicia Muñoz, Ornella Sotelo, Ximena Toledo

**Affiliations:** 1 Postgraduate Program in Orthodontics and Dentofacial Orthopedics, Faculty of Dentistry, University of Chile, Santiago, Chile; 2 Department of Pediatric and Dentofacial Orthopedics, Faculty of Dentistry, University of Chile Santiago, Chile

## Abstract

**Background:**

Orthognathic surgery (OS) corrects skeletal and dental discrepancies in the maxillofacial region, improving occlusion, masticatory function, respiration, and facial aesthetics. However, the immediate and intermediate postoperative periods are frequently associated with acute pain, facial edema, neurosensory disturbances (e.g., paresthesia of the inferior alveolar and/or infraorbital nerves), and restricted mandibular mobility (trismus), which may prolong recovery and impair quality of life. Physiotherapeutic rehabilitation has emerged as a key component of postoperative care, yet standardized guidance remains limited. The objective is to present a standardized, evidence-based physiotherapeutic protocol for managing common postoperative complications after OS, aiming to optimize functional recovery and reduce morbidity.

**Material and Methods:**

A narrative review of the literature on postoperative physiotherapeutic rehabilitation in OS was conducted, focusing on interventions for pain, edema, neurosensory disturbances, trismus, and bone healing. The protocol was developed within the Postgraduate Program in Orthodontics and Dentofacial Orthopedics, Faculty of Dentistry, University of Chile, integrating current evidence and clinical experience.

**Results:**

The protocol proposes multimodal strategies-including photobiomodulation, manual therapy, and therapeutic exercises-to address the most frequent postoperative complications. Evidence supports early, structured, and systematic intervention to improve analgesia, accelerate edema resolution, enhance neurosensory recovery, increase mandibular mobility, and promote bone healing.

**Conclusions:**

Early, structured physiotherapeutic management is fundamental to postoperative care in OS. The proposed protocol offers a replicable, evidence-based framework to facilitate optimal recovery and functional reintegration.

## Introduction

Orthognathic surgery (OS) is a complex surgical procedure indicated for the correction of skeletal and dental discrepancies of the maxillofacial region, with the objective of improving occlusion, masticatory function, respiration, and facial aesthetics. Despite usually favorable long-term outcomes, the immediate and intermediate postoperative periods are often associated with complications, most frequently acute pain, facial edema, temporary or permanent neurosensory disturbances -particularly paresthesia of the inferior alveolar and/or infraorbital nerves-, and restricted mandibular mobility (trismus). These sequelae may compromise patient comfort, prolong recovery time, and negatively affect quality of life.

Physiotherapeutic rehabilitation is essential in the management of these postoperative complications. An early, structured, and systematic intervention reduces morbidity and enhances functional outcomes (1 , 2). The presented protocol was developed within the Postgraduate Program in Orthodontics and Dentofacial Orthopedics at the Faculty of Dentistry, University of Chile, to provide a standardized, evidence-based guide for physiotherapeutic management in patients undergoing OS, aiming to optimize recovery and facilitate functional reintegration.

The efficacy of kinesitherapy in the postoperative management of OS is supported by multiple therapeutic modalities, targeting specific complications.

Photobiomodulation (LLLT)

Low-Level Laser Therapy (LLLT), also referred to as photobiomodulation (PBM), utilizes photons to stimulate cellular and tissue responses. Its mechanisms of action include increased ATP synthesis, nitric oxide release, enhanced blood flow, and modulation of intracellular signaling pathways that promote tissue repair and healing (3 , 4). LLLT exhibits a biphasic dose-response relationship, making accurate dosimetry essential for optimizing clinical outcomes (5).

Scientific evidence supports the use of LLLT in the postoperative phase of OS in the following domains:

- Pain and Edema: LLLT has demonstrated significant efficacy in reducing postoperative facial pain and swelling (6 , 7). Appropriate protocols enhance tissue responsiveness, attenuate inflammatory processes, and decrease patient-reported pain levels (7).

- Neurosensory Recovery: Sensory disturbances are a frequent postoperative concern. LLLT facilitates regeneration of affected nerve tissues, improving neurosensory recovery and reducing paresthesia, particularly in the medium and long term (8 , 9). Diode laser therapy has shown outstanding improvements in tactile sensitivity and pain perception, with superior outcomes when initiated early and applied at higher treatment frequencies (10 - 12).

- Bone Healing: In addition to its anti-inflammatory and analgesic properties, LLLT can enhance bone density and accelerate the repair of maxillofacial bone defects, contributing to improved healing (13).

Therapeutic Ultrasound

Therapeutic ultrasound, particularly in pulsed mode, has shown efficacy in managing postoperative complications (14). Low-Intensity Pulsed Ultrasound (LIPUS) is effective in controlling pain, inflammation, and wound healing after oral surgical procedures, contributing to the reduction of edema and trismus (15 , 16). Its effects are primarily mechanical, including micro-acoustic streaming and stable cavitation, which stimulate cellular activity, enhance blood flow, promote hematoma resorption, and improve scar tissue organization (17 , 18).

Transcutaneous Electrical Nerve Stimulation (TENS)

TENS is a non-invasive electroanalgesic modality for the management of acute and chronic pain (19). Its mechanism is based on the gate control theory, whereby stimulation of large-diameter afferent fibers inhibits nociceptive transmission, in addition to promoting endogenous endorphin release (19). In the postoperative context of OS, TENS can aid pain modulation, enhance neuromuscular function of masticatory and cervical muscles, improve patient comfort, and facilitate earlier initiation of therapeutic exercises (19 , 20).

Physiotherapy and Exercise Therapy

Physiotherapy plays a pivotal role in functional rehabilitation, with multimodal approaches combining various techniques demonstrating the greatest efficacy (21 , 22).

Mandibular Mobility: Early, structured physiotherapy supports recovery of mandibular range of motion and three-dimensional functional capacity (1 , 2).

Sensory Retraining: Targeted exercises can reduce long-term sensory disturbances (23). LLLT and LED therapy serve as effective adjuncts to neurosensory rehabilitation (21).

Edema Management: Lymphatic kinesio taping, a neuromuscular bandaging technique, has been shown to reduce postoperative inflammation by improving microcirculation of blood and lymph during the healing process (24 , 25). Manual lymphatic drainage, when combined with compression garments, has also demonstrated significant reductions in early postoperative edema of the head and neck region (26).

## Material and Methods

This narrative review integrates updated scientific literature (2010-2025) with the clinical experience systematized by Kinesiologist Alejandro Fernández de la Reguera within the Postgraduate Program in Orthodontics and Dentofacial Orthopedics, Faculty of Dentistry, University of Chile.

## Results

Description of the Physiotherapeutic Protocol

The protocol is structured into preoperative and postoperative phases, aimed at preparing the patient and optimizing functional recovery.

Preoperative Phase

Duration: 5-7 days before surgery.

Objectives: To prepare soft tissues and musculature for the intervention, to maintain mandibular range of motion, and to educate the patient on postoperative care.

Components

Soft tissue elongation (lips, cheeks, buccinatopharyngeal fascia) to improve flexibility and reduce tension.

Massage of the Mandibular Musculature.

**Figure 1:**
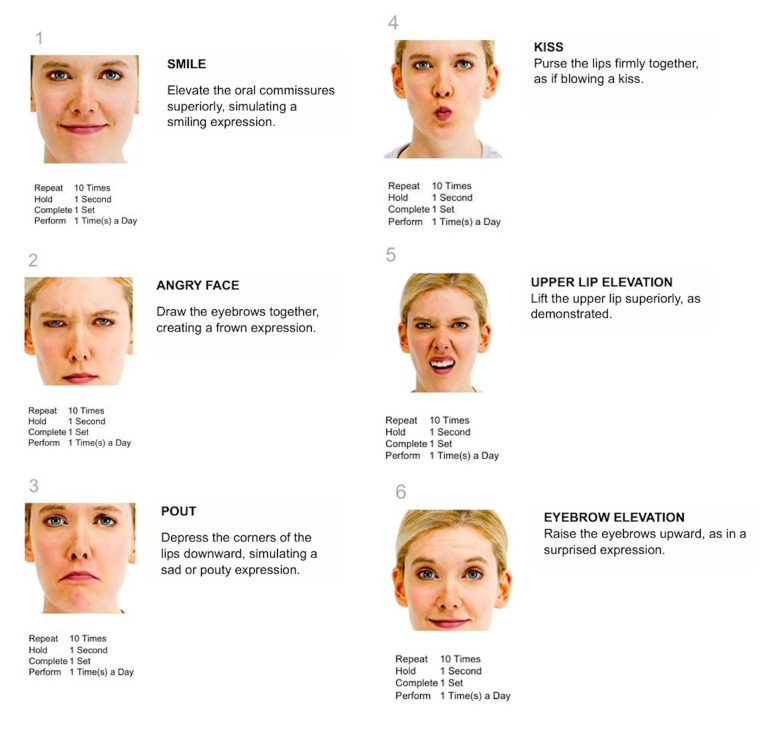
Facial mimic exercises.

Facial mimic exercises: Activation routine including smile, angry face, pout, kiss, and elevation of upper lip and eyebrows (Figure 1); 10 repetitions per exercise, once daily.

1

Mandibular joint mobility: Active mouth opening and lateral movement exercises to preserve preoperative range of motion.

Manual lymphatic drainage: Cervical and facial lymph nodes pumping techniques to stimulate the lymphatic circulation.

Preoperative education: Guidance on postoperative care, application of local cold, oral hygiene, soft diet adherence, and postural recommendations to minimize edema and discomfort.

Postoperative Phase

Initiation: Postoperative day 4-5, once acute inflammation begins to subside.

Objectives: To reduce inflammation, to modulate pain, to prevent fibrosis, to progressively restore neuromuscular function, and to recover mandibular opening and coordination.

Components:

Manual lymphatic drainage: Once or twice daily until 9-12 days postoperatively.

Lymphatic kinesio taping: Applied from day 0, replaced on days 3 and 5, maintained for one week.

Myofascial induction: Release of tension points in the masseter, temporalis, temporalis tendon, and lateral pterygoid muscles.

Cervical elongation: Gentle stretching of posterior cervical and scalene muscles to release compensatory tension and improve posture.

Mandibular opening exercises: Progression from opening pinch, joint distraction, isometric opening, condylar rotation, to lateral movements.

Photobiomodulation (LLLT): Intraoral and/or extraoral application (600-940nm), focused on osteotomy sites and inferior alveolar nerve pathway to promote bone healing, reduce edema, modulate pain, and accelerate neurosensory recovery.

Transcutaneous Electrical Nerve Stimulation (TENS): Used in early postoperative phases to reduce pain and enhance neuromuscular function of masticatory and cervical muscles; 20-30 min sessions, 1-2 times per week, according to tolerance.

Therapeutic ultrasound: Pulsed mode (1 MHz, 0.5 W/cm²) in early phases for hematoma and edema resolution; later, continuous 3 MHz mode for scar remodeling and flexibility.

Facial mimic reinforcement: Daily exercises to maintain muscle tone, fine motor control, and expressiveness.

Protocol Progression

Week 1: Manual lymphatic drainage, LLLT, TENS, gentle mobilization, and pulsed ultrasound for hematoma management.

Week 2: Gradual increase in joint range of motion and intensification of myofascial induction.

Week 3: Introduction of mandibular and cervical stabilization exercises; ultrasound for scar management.

Follow-up: Kinesiological monitoring up to 6 months postoperatively, adapting the protocol to individual progress.

## Discussion

This physiotherapeutic protocol for the postoperative management of OS integrates multiple therapeutic modalities with the objective of achieving complete functional recovery and minimizing complications. The inclusion of LLLT is supported by evidence demonstrating its effectiveness in reducing pain and edema (6 , 7), accelerating neurosensory recovery (9 - 12), and enhancing bone healing (13). The parameters proposed in this protocol are consistent with those reported in the literature for maxillofacial applications.

Therapeutic ultrasound, particularly in pulsed mode, plays a key role in the early management of edema and hematomas (14 , 15), and later contributes to scar remodeling through biomechanical effects that improve tissue organization, as demonstrated in studies of postoperative oral surgery (16 - 18).

TENS is incorporated as a pain management strategy during the initial postoperative phase, enhancing patient comfort and enabling more active participation in rehabilitation (19). Although high-quality evidence specifically in OS is limited, TENS is widely recognized in musculoskeletal rehabilitation for symptomatic pain relief (19 , 20).

Physiotherapeutic exercises and manual techniques-including lymphatic drainage, kinesio taping, and myofascial induction-form the core of the protocol. These approaches have been shown to improve mandibular range of motion, to reduce facial edema, and to facilitate neuromuscular re-education (1 , 2 , 23 - 26). The structured progression of the protocol allows gradual adaptation, optimizing recovery while avoiding overload of healing tissues. The preoperative phase is particularly important, as patient and tissue preparation can positively influence postoperative outcomes.

Implementation of this standardized protocol, developed by the Faculty of Dentistry, University of Chile, provides a consistent, evidence-based framework for clinicians, promoting predictable outcomes and enhancing quality of life for patients undergoing OS. Nonetheless, further research, including controlled clinical trials, is necessary to validate its efficacy in larger patient cohorts.

## Conclusions

The Multimodal Physiotherapeutic Protocol for the Management of Postoperative Complications in OS, developed by the Faculty of Dentistry, University of Chile, represents a comprehensive and evidence-based approach, by combining physiotherapy techniques, targeted exercises, and modalities such as LLLT, therapeutic ultrasound, and TENS, the protocol aims to optimize functional recovery, to reduce postoperative complications, and to enhance patient experience. This protocol provides a foundation for standardized postoperative rehabilitation in OS and serves as a reference for clinicians and future multicentric clinical studies.

## Data Availability

Declared None.
